# *Termitotrox cupido* sp. n. (Coleoptera, Scarabaeidae), a new termitophilous scarab species from the Indo-Chinese subregion, associated with *Hypotermes* termites

**DOI:** 10.3897/zookeys.254.4285

**Published:** 2012-12-21

**Authors:** Munetoshi Maruyama

**Affiliations:** 1 The Kyushu University Museum, Hakozaki 6-10-1, Higashi-ku, Fukuoka, 812-8581 Japan

**Keywords:** Termitophily, Termitotroginae, Termitotrogini, new species, Indo-Chinese subregion, smallest scarab

## Abstract

*Termitotrox cupido* **sp. n.** is described from Cambodia and represents the first discovery of *Termitotrox* Reichensperger, 1915 from the Indo-Chinese subregion of the Oriental region. The type series was collected from fungus garden cells of *Hypotermes makhamensis* Ahmad, 1965 (Isoptera, Termitidae, Macrotermitinae). *Hypotermes* Holmgren, 1917 was previously an unknown host of *Termitotrox* species. The new species is readily distinguished from all known congeners by having wing-shaped trichomes on the elytra and is most probably the world’s smallest scarab, at 1.2 mm in length.

## Introduction

The scarab genus *Termitotrox* Reichensperger, 1915 is composed of ten blind, flightless species from the Ethiopian region (eight species) and the Indian subregion of the Oriental region (two species) (Krikken 2008). Wasmann (1918) originally established the tribe Termitotrogini in the subfamily Coprinae of Scarabaeidae to include this genus, but the rank of the taxon was treated differently by different authors from a distinct family of Scarabaeoidea (Termitotrogidae: Tangelder and Krikken 1982; Dellacasa 1988), a subfamily of Scarabaeidae (Termitotroginae: Smith 2006; Bouchard et al.2011), to a tribe of Aphodiinae (Termitotrogini: Scholtz and Grebennikov 2006). Currently, the subfamily status or the tribal status is accepted, but it remains unstable until a precise phylogenetic position of *Termitotrox* within Scarabaeidae is investigated. Most species have been found in fungus gardens of the fungus-growing termite genera *Odontotermes* Holmgren, 1912 or *Protermes* Holmgren, 1910 (Isoptera, Termitidae, Macrotermitinae). Recently, I collected an extraordinary, undescribed species of *Termitotrox* in Cambodia which is far from the known localities of the genus in the Oriental region (India) and is the first discovery of the genus in the Indo-Chinese subregion. It was collected from fungus garden cells of *Hypotermes* Holmgren, 1917 (also Macrotermitinae), a previously unknown host. This paper describes it as a new species.

When Arrow (1920) described *Termitotrox minutus* (originally in the genus *Aphodiocopris* Arrow, 1920) from India, he stated “probably the smallest Lamellicorn beetle [= scarab] hitherto discovered”. *Termitotrox minutus* is approximately 1.5 mm in length. Subsequently Pittino (2007) described *Microtermitodius atomus*, ranging from 1.4 to 1.7 mm, which reduced the size of the smallest known scarab still further. However, the new species is 1.2 mm long, and is therefore likely the world’s smallest scarab found thus far.

## Materials and methods

In August 2012, I visited Angkor Wat, Siem Reap, Cambodia, a typical tropical monsoon forest, for five days and worked a total of 15 hours collecting termitophiles associated with fungus-growing termites. I examined fungus gardens of the genera *Macrotermes* Holmgren, 1910 (four species), *Odontotermes* (three species), *Microtermes* Wasmann, 1902 (one species) and *Hypotermes* (one species). I found 25 *Termitotrox* beetles from fungus garden cells of *Hypotermes makhamensis* Ahmad, 1965 by examining more than 80 fungus gardens. The beetles were put in a killing tube (35 ml) with tissue paper and ethyl acetate; two hours later they were removed from the tube and kept in 80% ethanol. Twenty two specimens were dried and mounted for morphological observation, and remaining three are kept in 99% ethanol for future DNA extraction. Specimen photographs were taken using a Canon EOS 60D with a Canon MP-E 65 mm 1-5× macro lens and mounted using a software CombineZM. Images of living beetle were taken by the same camera set in the field by Dr. Takashi Komatsu. Terminology of the species description follows Krikken (2008). The type series is deposited in the author’s collection in the Kyushu University Museum, Fukuoka (KUM); Canadian Museum of Nature, Ottawa (CMN); The Natural History Museum, London (NHM); and the University of Nebraska State Museum (UNSM).

## Taxonomy

### Genus Termitotrox

Reichensperger

Termitotrox Reichensperger 1915: 16 (type species: *Termitotrox consobrinus* Reichensperger, 1915, by monotypy).
Aphodiocopris Arrow 1920: 432 (type species: *Aphodiocopris minutus* Arrow, 1920, by monotypy).

#### Comments.

See Krikken (2008) for generic review.

### Termitotrox cupido

Maruyama sp. n.

urn:lsid:zoobank.org:act:03F2CD83-2D9D-4F22-8CFE-0CF88264AA2C

http://species-id.net/wiki/Termitotrox_cupido

#### Type materials.

Holotype female, north of Preah Khan, Siem Reap, Cambodia, 19 VIII 2012, M. Maruyama (KUM). Paratypes, 6 males, 10 females, same data as holotype (KUM, NHM); 4 males, 1 females, 3 unsexed, same data but 21 VIII 2012 (CMN, KUM, UNSM).

#### Etymology.

*Cupido* is the god of desire and love in Roman mythology and is often illustrated as a small, winged boy. The new species is named in reference to the wing-shaped trichomes on the elytra and the remarkably small body size. Noun in apposition.

#### Diagnosis.

This species is probably related to *Termitotrox minutus* (Arrow, 1920) because of its small body size and shape of elytra but easily distinguished from it by the spherical elytra, the presence of the trichomes on the elytra and the smaller body.

#### Description of holotype female.

General colour uniformly reddish brown, slightly matt; length 1.21 mm. ***Head*.** Surface generally evenly convex, with only a slight callosity at clypeofrontal transition. Lateral margin of head entirely, finely marginate. Clypeal outline evenly rounded over entire length. Clypeofrons reddish brown, glabrous, distinctly, moderately punctate; vertex with deep groove medially, and 6 or 7 pairs of sharply defined, elongate primary punctures. Clypeofrontal border at (vague) suture straight; genal tip obtusely angular (in dorsal view); genal surface depressed. Antennal club yellowish brown. ***Prothorax*.** Prothorax reddish brown, narrower than elytra, sides (in dorsal view) evenly rounded over anterior half. Emargination at center apex not margined, anterolateral lobe rounded, edge slightly projecting downward (forming side of anterolateral propectoral cavity). Pronotal sides steeply declivous. Posterolateral section of pronotum rounded. Basolateral areas concave, with 1 feeble ridge around base; asymmetrical, left part with a tubercule near base. Apical lobe asymmetrical, with right side near apex roundly emarginate. Pronotal surface glabrous. Costae densely punctate, broader intercostal sulci with distinct, longitudinal wrinkles. Discal depression deep; surface, apart from some local micropunctation, smooth. Pronotal pattern of longitudinal costae as follows: Median costa indistinct around apical 1/5; basomedian section narrow, surface deplanate, shallowly concave. Central depression posterolaterally delimited by depressed area of paramedian costa. Paramedian costa anteriorly broad, distinct, continuing to about half of pronotal length. Sublateral costa narrow, distinct, tapering posteriad to about half of pronotal length, reaching paramedian costa. Lateral costa anteriorly broad, distinct, extending from anterolateral lobe caudad, tapering to base of pronotum. Marginal costa posteriorly broad, ending at depressed basolateral area. ***Elytra*.**Semi-spherical, reddish brown, matt, with 6 interstrial costae and intervening striae, and with trichomes at base of costae 2–6 to form wing-shaped patches. Humeral and apical elytral umbones absent; apicosutural edge nearly rectangular, slightly protruding. Epipleuron wide. Elytral striae distinct, deeply impressed, with transverse, weak costae from base to apex to form quadrate cells; striae 1 and 2 reaching basal half. Discal interstrial costae broadly trapezoidal (in cross-section), surface with dense, scattered punctures. Elytral pattern of interstrial costae as follows: costa 1 (next to suture) narrow, shiny, almost rectilinear; costa 2 shiny, tapering in front, stopping at basal half. Costa 3 complete, slightly narrowed at middle. Costae 4–6 complete, strongly developed, Costa 7, 8 and 9 apparently fused together. Anterolateral part of propectus deeply excavate. Preprosternal apophysis distinct, with several setae. Remainder of propectus glabrous, dark reddish brown. Posterolateral area of propectus with some ridges and grooves. Postprosternal surface with small, shallow, median impression. Transverse mesometasternal groove between posterior edges of mesocoxae distinct. ***Mesothorax*.** Mesosternum with a pair of identical, question-mark shaped grooves bordering the mesocoxae ; mesosternal surface reddish brown, glabrous, flattened; anterior surface densely micropunctate. ***Metathorax*.** Metasternum evenly convex, glabrous, and with fine perimarginal groove all around; reddish brown, infuscate laterally. ***Abdomen*.** Venter with 5 visible sternites, all reddish brown, matt, without grooves, sparsely micropunctate. Pygidium reddish brown, glabrous, base broadly margined; surface generally convex; surface lacking distinct microsculpture, sparsely micropunctate. ***Legs*.** Procoxa protuberant. Profemur brown, underside glabrous, sparsely micropunctate; outline broadly elliptic, emarginate distally. Protibia pale brown, broad, with short setae, microsculpture weak; shape strongly complanate, with 2 external denticles, no basal serration; apex straight, transverse, with distinct apico-internal spine; internal side strongly dilated from slender base. Protarsus twice longer than width of tibial apex, slender, yellowish; segment 1 inserted in fine groove, as long as segments 2–4 combined. Mesocoxae reddish brown, widely separated, slightly divergent anteriad. Mesofemora brown, broadly elliptic in outline, distally emarginate, surface moderately micropunctate, glabrous. Mesotibiae reddish brown, with several setae, broad, dilated near base, nearly parallel-sided from basal half to apex, edges entire; tibial apex deeply emarginate, with pair of acuminate apico-internal spurs, external one long, slightly curved, internal one short, straight; upper side of mesotibiae with fine longitudinal ridge near outer edge, underside with fine sinuate ridge from base to apico-internal section; with long setae around apical 2/5. Metatibiae similar to mesotibiae, but gently dilated apicad, with apex shallowly emarginate. Meso- and metatarsi brown, compacted-complanate, segments 1–4 short. Length of inner apical spur of metatibia 1/4 of metatibia, reaching base of tarsal segment 5.

#### Male.

Aedeagus (Figs 4, 5) large, half as long as body length; paramere half as long as phallobase, gently narrowed apicad, curved near truncate apex.

**Figures 1–3.**
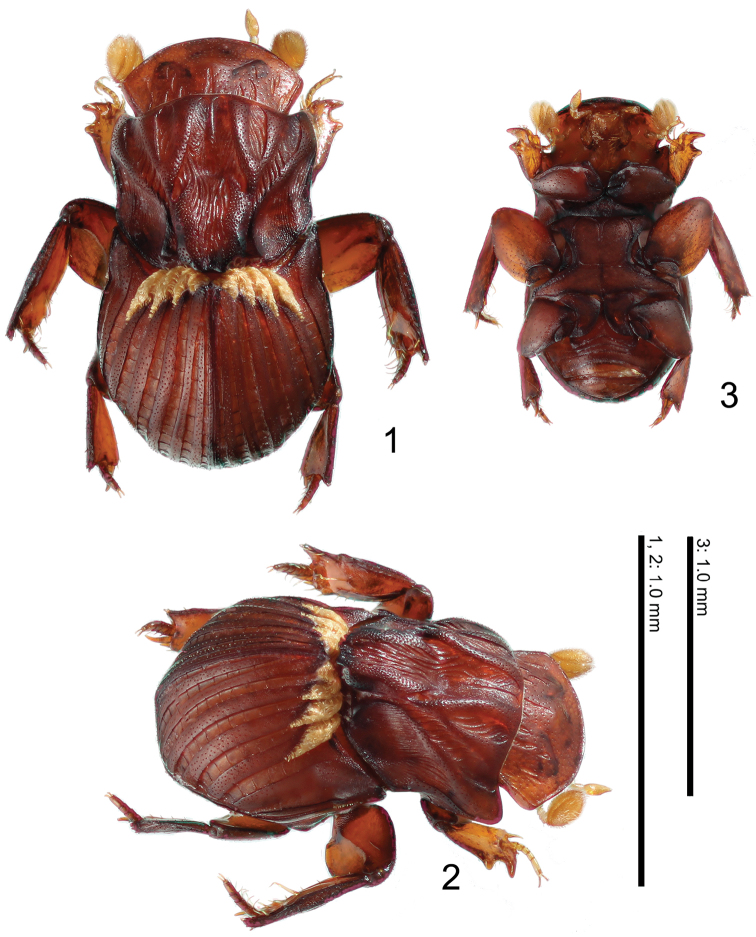
Habitus of *Termitotrox cupido* sp. n. **1** Holotype, dorsal view **2** same, antero-lateral view **3** paratype, ventral view

**Figures 4–6.**
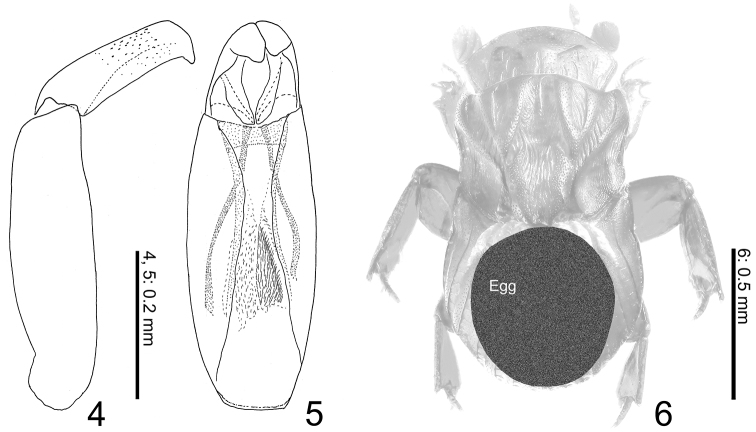
*Termitotrox cupido* sp. n. **4** Aedeagus, lateral view **5** same, ventral view **6** mature egg (shaded circle) inside of female body.

#### Variations.

Apical lobe of pronotum variable in shape, sometimes symmetrical, gently rounded at apex. Costa 7 of elytra sometimes indistinct.

#### Measurements in mm.

Body length 1.13–1.22; maximum width of head 0.48–0.52; median dorsal length of pronotum 0.47–0.51, maximum width 0.54–0.59, sutural length of elytron 0.50–0.54, maximum width 0.62–0.67.

#### Symbiotic host.

*Hypotermes makhamensis* (determined by Dr. Yoko Takematsu).

#### Remarks.

No significant sexual dimorphism is detected. Male aedeagus is large compared with its body size. Female ovary contained a single huge egg occupying the greater part of the abdomen and metathorax (Fig. 6).

## Discussion

**Figure 7.**
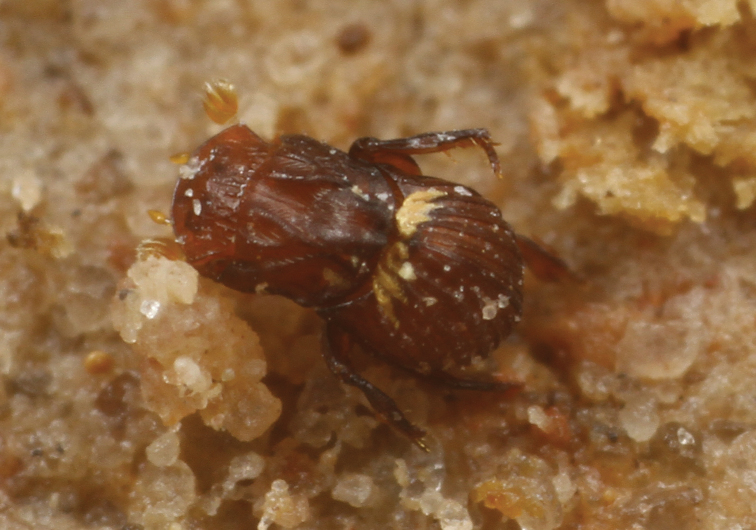
*Termitotrox cupido* sp. n. walking on a fungus garden cell.

**Figure 8.**
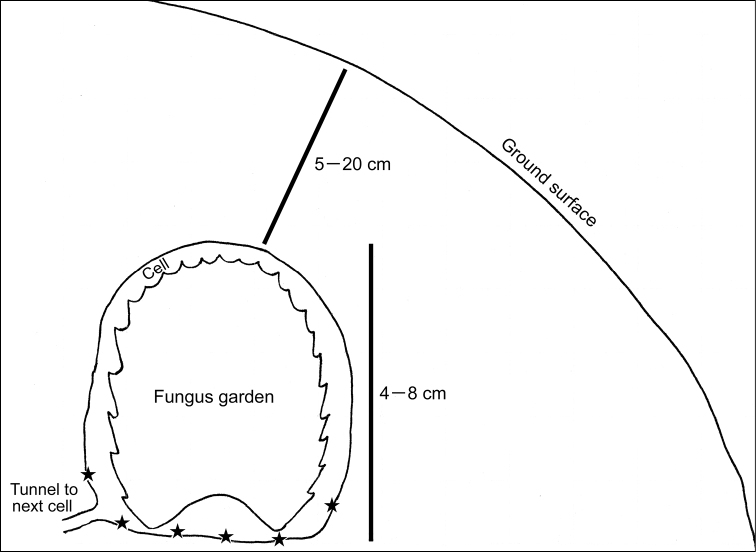
A schematic illustration of a fungus garden cell of *Hypotermes makhamensis* and places where *Termitotrox cupido* beetles were found (black stars).

*Termitotrox cupido* specimens were found only on the walls of the fungus garden cells of *Hypotermes makhamensis* (Figs 7, 8), unlike many other termitophiles associated with fungus-growing termites, which are usually found inside the fungus gardens themselves. In the same habitat (on the cell wall), undescribed species of *Odontoxenia* Schmitz, 1915, *Clitelloxenia* Kemner, 1932, *Ridiculiphora* Disney, 1997 (Diptera, Phoridae) and *Discoxenus* Wasmann, 1904 (Coleoptera, Staphylinidae) were found, but they were also found inside the fungus gardens.

When the fungus garden was removed from the cell, the beetles walked slowly on the cell wall to escape to a tunnel connected to the other fungus garden cells, by following the termites. No direct contact with the beetle by the termites was observed even though the beetle walked among highly dense columns of the termites. Since *Termitotrox cupido* is so small (≈ 1.2 mm) and the termites were rushing to escape the disturbance, further observations of its termite association were not possible.

No behavioural information of *Termitotrox* species is available, other than that reported here for *Termitotrox cupido*. In two species of Corythoderini, the other termitophilous scarab tribe associated with fungus growing termites, beetles were being carried by worker termites as they do for their own nymphs (Kistner 1982; Maruyama 2012): the worker termite grabbed a handgrip-like structure which is formed by the median lobe of the pronotal base (medially projected posterior margin) and a median projection of the elytral base of the beetles. Though *Termitotrox* species do not possess an elytral median projection, they share the pronotal median lobe which is well developed and is handgrip-shaped, hence it could be hypothesized that *Termitotrox* beetles are likewise carried by the worker termites.

*Termitotrox cupido* is characterized by the large trichomes on the elytral costae, and these trichomes are not known among other *Termitotrox* species. Among inquilinous Aphodiinae trichomes on body surfaces have been observed in members of the tribes Corythoderini, Termitoderini, Stereomerini, and Rhyparini. Wasmann (1903) observed secretory tissue in *Chaetopisthes assmuthi* Wasmann 1911 (Corythoderini) under the posterior part of the pronotum and apices of the elytra, where the trichomes originate. This kind of tissue could exist in the trichomes of other species, and it probably produces chemical signals that target the host termites. Krikken (2008) speculated that the body surface convexities (including elytral costae) in the other *Termitotrox* species that do not possess trichomes might be filled with glandular tissue. The trichomes on the elytral costae in *Termitotrox cupido* indicate the presence of glandular tissue in the convexities of the elytral costae, suggesting glands might by present in this body region in the other species.

In the Oriental region the known distribution of *Termitotrox* species was restricted to the Indian subregion before the present finding of *Termitotrox cupido* in Cambodia in the Indo-Chinese subregion. Krikken (2008) mentioned that the Indo-Afrotropical distribution of *Termitotrox* is analogous to that of the Corythoderini. Recently, I collected a new genus and species of Corythoderini in the same place as *Termitotrox cupido* in Cambodia (Maruyama 2012). Aanen & Eggleton (2005) suggested that fungus-growing termites originated in the African rainforest, and their distributional range subsequently extended from tropical Africa to Southeast Asia. Corythoderini and *Termitotrox* might also have originated in Africa and have extended their distributions along with their host termites.

## Supplementary Material

XML Treatment for Termitotrox

XML Treatment for Termitotrox cupido
